# Cnidofest 2018: the future is bright for cnidarian research

**DOI:** 10.1186/s13227-019-0134-5

**Published:** 2019-09-04

**Authors:** Shuonan He, Juris A. Grasis, Matthew L. Nicotra, Celina E. Juliano, Christine E. Schnitzler

**Affiliations:** 1 Stowers Institute for Medical Research, Kansas City, MO 64110 USA; 2 School of Natural Sciences, University of California, Merced, CA 95343 USA; 3 Department of Surgery, Thomas E. Starzl Transplantation Institute, University of Pittsburgh, Pittsburgh, PA 15261 USA; 4 Pittsburgh Center for Evolutionary Biology and Medicine, University of Pittsburgh, Pittsburgh, PA USA; 5 Department of Immunology, University of Pittsburgh, Pittsburgh, PA USA; 6 Department of Molecular and Cellular Biology, University of California, Davis, CA 95616 USA; 7 Whitney Laboratory for Marine Bioscience, University of Florida, St. Augustine, FL 32080 USA; 8 Department of Biology, University of Florida, Gainesville, FL 32611 USA

**Keywords:** Cnidarians, *Hydra*, *Hydractinia*, *Nematostella*, *Aiptasia*, *Cassiopeia*

## Abstract

The 2018 Cnidarian Model Systems Meeting (Cnidofest) was held September 6–9th at the University of Florida Whitney Laboratory for Marine Bioscience in St. Augustine, FL. Cnidofest 2018, which built upon the momentum of Hydroidfest 2016, brought together research communities working on a broad spectrum of cnidarian organisms from North America and around the world. Meeting talks covered diverse aspects of cnidarian biology, with sessions focused on genomics, development, neurobiology, immunology, symbiosis, ecology, and evolution. In addition to interesting biology, Cnidofest also emphasized the advancement of modern research techniques. Invited technology speakers showcased the power of microfluidics and single-cell transcriptomics and demonstrated their application in cnidarian models. In this report, we provide an overview of the exciting research that was presented at the meeting and discuss opportunities for future research.

## Introduction

Cnidaria (corals, jellyfish, sea anemones, and hydroids) is a phylum of aquatic animals, unified by the presence of specialized stinging cells called cnidocytes [1]. These beautiful and exotic creatures have fascinated biologists since the dawn of experimental biology [2, 3]. Their phylogenic position as the sister group to Bilateria makes them key to addressing long-standing questions regarding animal relationships and evolution [4–7]. Until recently, cnidarian research has been hindered by the lack of advanced molecular and genetic approaches. However, rapidly advancing technologies, including genome sequencing and gene-editing tools, are now being applied to many cnidarian species. Large collections of genomic and transcriptomic data of diverse cnidarian species have now been generated [8–12] and single-cell sequencing technologies are deepening our understanding of cnidarian development and evolution [13, 14]. These data, in combination with new gene-editing capabilities [15–17], are opening new experimental avenues and enabling the use of many different cnidarians to address a myriad of biological questions.

**Fig. 1:**
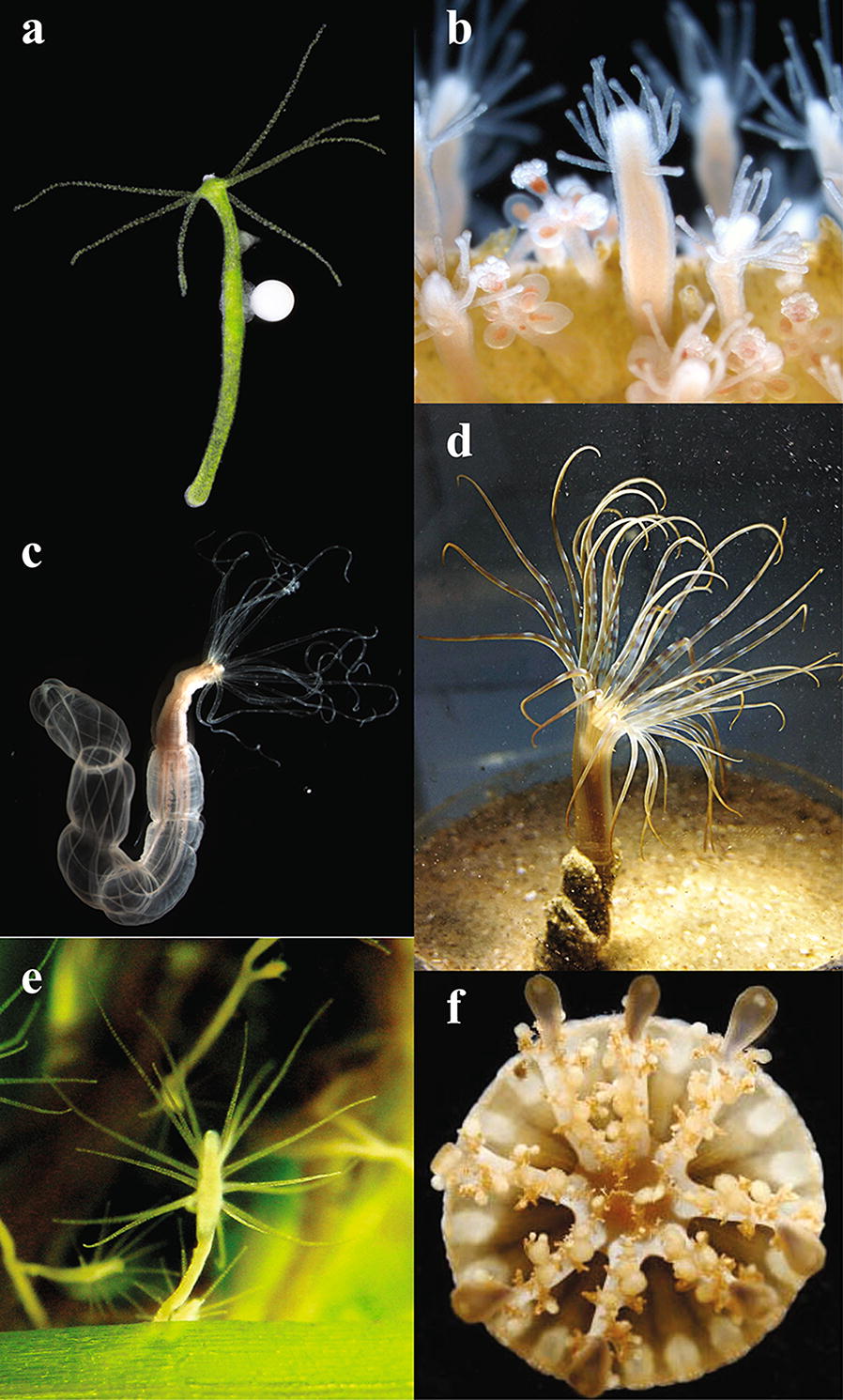
Cnidarian model systems represented at Cnidofest. **a** A hermaphroditic green *Hydra* polyp bearing a testes and an egg (courtesy of Stefan Siebert). **b** Gastrozooids and gonozooids of a *Hydractinia symbiolongicarpus* colony (courtesy of Steven Sanders). **c** *Nematostella vectensis* adult polyp (courtesy of Shuonan He). **d** The banded tube-dwelling anemone, *Isarachnanthus nocturnes* (courtesy of Sérgio Stampar). **e** A *Cordylophora* colony growing on eelgrass (courtesy of Nadine Folino Rorem and E. Sally Chang). **f** Medusa of *Cassiopea* spp. (courtesy of Casandra Newkirk)

Cnidofest 2018 (http://www.cnidarianmodelmeeting.org) was organized to foster the expansion of the cnidarian research community and emphasize the application of modern molecular tools to both classic and emerging cnidarian models. As a successor to the Hydroidfest 2016 meeting [18], which emphasized hydrozoan research, Cnidofest 2018 widened its focus to include representatives from many cnidarian taxa. Two established model organisms, *Hydra* and *Nematostella*, appeared in approximately 60% of the abstracts. However, the overall meeting agenda was diverse, with 11 cnidarian species featured during 44 oral presentation sessions and a total of 22 species represented by the 85 submitted abstracts. Nearly all major cnidarian clades were represented (Fig. 1), including traditionally underrepresented groups, such as Cubozoa, Staurozoa, and Myxozoa. Newly sequenced genomes and transcriptomes are giving these lesser known, yet fascinating animals a significant boost.

A major goal of the Cnidofest 2018 meeting was to contribute to the career development of trainees in the cnidarian research community. With funding support from the National Science Foundation and the University of Florida Office of Research, registration fees were waived for nearly all trainees and travel allowances were awarded to 16 domestic and 5 international trainees. This allowed for trainees to make up nearly 70% of the total attendance, and 68% of the oral presentations (30/44) were given by students and postdocs. The environment provided at Cnidofest enabled students and postdocs to showcase their work and interact directly with established researchers in this small, yet vibrant community. With these fantastic young scientists, new species, new technologies, and new ideas, the cnidarian research community is growing quickly and the future is bright. In this report, we provide an overview of the exciting research presented at Cnidofest 2018.

## Keynote address: cnidarian symbionts and the fate of coral reefs

Virginia Weis (Oregon State University) is an inspiring figure in the cnidarian research community. For more than two decades, she has pioneered and promoted coral symbiosis studies using *Aiptasia*, which is colonized by dinoflagellates from the family *Symbiodiniaceae* [19], as a model organism. Work from her lab and her collaborators successfully pushed forward our understanding of host–symbiont interactions on the molecular and cellular level. As the keynote speaker of Cnidofest 2018, Weis shared with the audience highlights covering 22 years of research done by her laboratory, her success in promoting and connecting the *Aiptasia* community, her vision of the future of symbiosis research, and her concern over global warming and the accelerating rate of coral extinction.

Weis emphasized the importance of translating findings in basic research into valuable tools for conservation biology. The lectin/glycan interaction and the complement pathway were identified as key players mediating host–symbiont recognition; disruption of these pathways blocks symbiont colonization in *Aiptasia* larvae. Based on these discoveries, Weis and her collaborators are currently testing symbionts with chemically modified glycans as an attempt to increase their colonizing abilities, with the ultimate goal of rescuing bleached coral populations. Meanwhile, by incubating *Aiptasia* with high concentrations of thrombospondin type 1 repeat (TSR) peptides, a key component of the complement pathway, Weis and colleagues turned normal symbionts into ‘super colonizers’. These approaches, though preliminary, may help to alleviate challenges in coral conservation. Finally, Weis laid out her vision for a community-wide effort to develop gene manipulation techniques such as CRISPR/Cas9 in both *Aiptasia* and their dinoflagellate symbionts. Thanks to the efforts of Weis, many well-established biologists such as John Pringle (Stanford) and Thomas Gilmore (Boston University) have adopted *Aiptasia* as a research organism in their own labs. Meanwhile, a younger generation of *Aiptasia* biologists are emerging with talent and dedication, highlighted by the exciting oral presentations and posters during this meeting. Emphasizing the need for multiple communities to unite around the complex issue of saving coral reefs, Weis stressed that “it takes a village to save corals…and we must take action before it’s too late.”

## Genomics: expanding genomic resources for cnidarian research

**Fig. 2:**
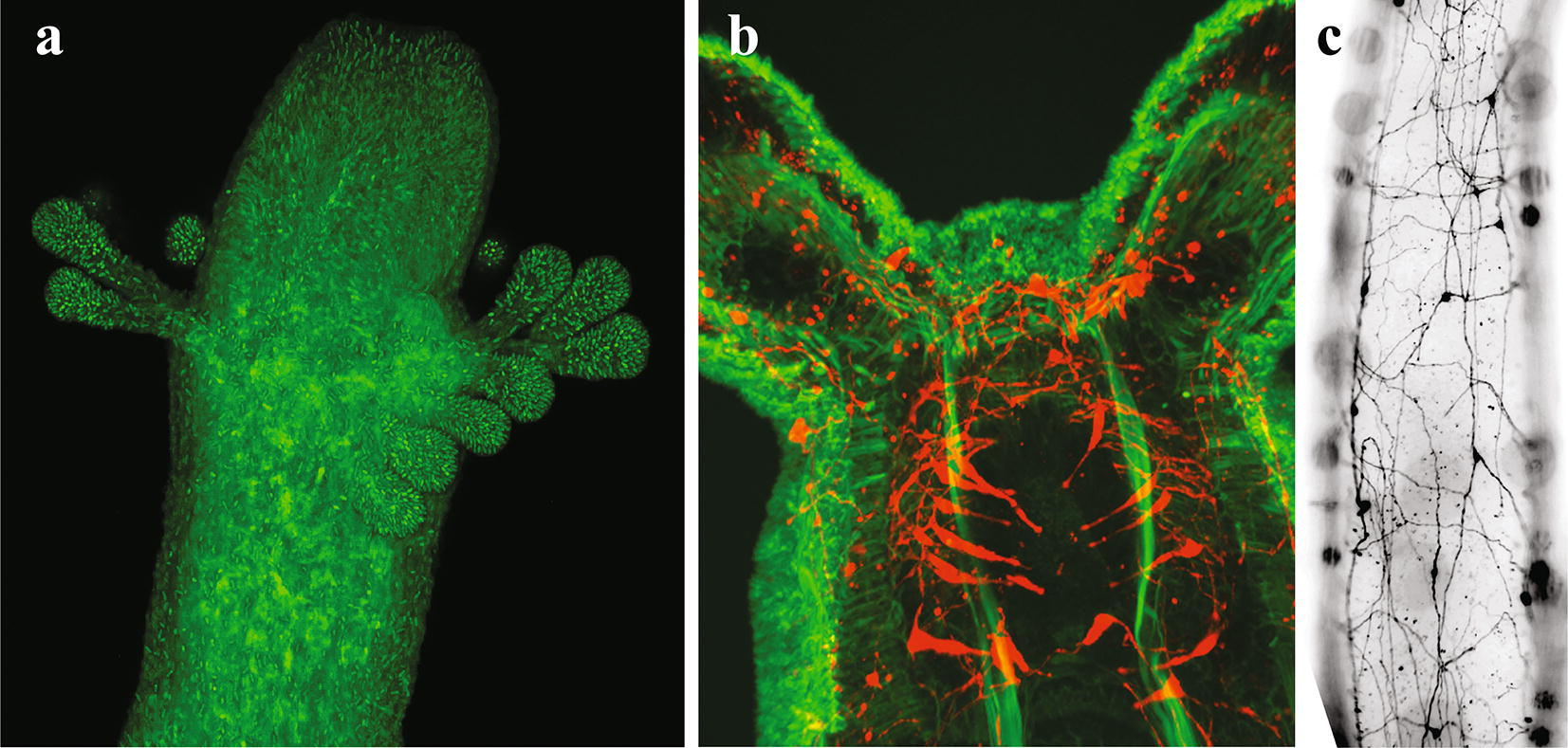
Technical advancements boost cnidarian research. **a** Fluorescent micrograph of a *Hydractinia symbiolonigcarpus* gastrozooid expressing eGFP (green) from the endogenous Eukaryotic elongation factor 1 alpha (*Eef1a*) locus. CRISPR/Cas9-mediated homologous recombination enables efficient and precise genome editing in *Hydractinia* and opens up new possibilities for future research (courtesy of Steven Sanders). **b** LWamide-positive neurons (red) surrounding the pharynx of a *Nematostella* primary polyp, counter stained with phalloidin (green). **c** LWamide-positive neurons in an adult *Nematostella* tentacle. Establishment of various transgenic lines such as NvLWamide:: mCherry animals illustrated above enables direct visualization of specific neurons and provides new insights into the development, regeneration, and function of the relatively diffused cnidarian nervous system (courtesy of Jamie Havrilak)

Cnidarian genomes hold a key to understanding animal phylogenetic relationships and provide the framework for exploring the evolution of complex biological processes from embryogenesis to aging. The opening session of Cnidofest 2018 included exciting advancements in comparative genomics and the development of genome engineering tools. Meeting host Christine Schnitzler (Whitney Laboratory for Marine Bioscience, University of Florida) presented genome and transcriptome assemblies of two *Hydractinia* species, *H. echinata*, and *H. symbiolongicarpus*. These colonial hydrozoans have long attracted attention from cnidarian researchers due to their regenerative and allorecognition capabilities [20–23]. Schnitzler is using a comparative genomics approach to shed light on the evolution of biological novelties. In addition, Schnitzler presented her latest efforts in collaboration with the Baxevanis group at NIH to establish and update publicly available *Hydra* and *Hydractinia* genome portals (https://research.nhgri.nih.gov/hydra/; https://research.nhgri.nih.gov/hydractinia), which continue to benefit the cnidarian research community. Wai Yee Wong (graduate student, University of Vienna) discussed the high percentage (> 50%) of transposable elements (TEs) found in the *Hydra* genome; the biological consequence of these TEs is unclear. Wong refined the TE annotations for *Hydra* using a novel analytic pipeline, RepeatCraft, and then performed comparative analyses across four Brown *Hydra* species and one Green *Hydra* species to ask how the expansion of TEs increased the genome sizes of Brown *Hydra* compared to Green *Hydra* [11, 24]. Wong found that one LINE family transposable element is significantly enriched in the Brown *Hydra* species as compared to the Green *Hydra*. Wong hypothesized that this lineage-specific LINE family TE expansion could partially explain the drastic genome size increase in Brown *Hydra*. Joseph Ryan (Whitney Laboratory for Marine Bioscience, University of Florida) discussed his efforts to refine phylogenetic relationships within Cnidaria by first constructing a “backbone” phylogenetic tree from 110 species followed by surveying the 18S rRNA sequences of thousands of cnidarian species to add as “leaves” on the tree. This large phylogenetic dataset includes many new taxa and promises to shed light on the positioning of some ambiguous lineages. Ryan proposed to use machine learning to extract biological traits from the literature as a method to further refine the cnidarian tree of life in a trait-based manner. Finally, Steven Sanders (postdoctoral researcher, University of Pittsburg) discussed his successful application of CRISPR/Cas9 genome-editing techniques in *Hydractinia.* As a proof-of-principle for gene knock-in, Sanders used homology-directed repair to insert GFP into the genome at the elongation factor 1 alpha (*Ef1a*) locus to create a transgenic line with stable expression of an EF1a–GFP fusion protein (Fig. 2a, [17]). Sanders ultimately plans to use gene editing to tag genes involved in allorecognition. Eviatar Weizman (graduate student, Bar-Ilan University) investigated the chromatin dynamics of *Nematostella* when adapting to an ever-changing environment. Weizman performed ATAC-seq, which assays genome accessibility, in adult animals subjected to two different light regimens. Pairing the ATAC-seq dataset with previously published RNA-seq results [25], he was able to demonstrate that transcription rhythmicity of key circadian genes correlates with the oscillation of chromatin accessibility through the light–dark cycle.

## Developmental biology: old genes play new tricks in cnidarians

Despite their distinctive morphology, cnidarians share many key developmental and disease-related genes with bilaterian animals. In two consecutive sessions centered around cnidarian developmental biology, eight speakers showcased the power of using cutting-edge techniques to address the function of evolutionarily conserved genes and signaling pathways. Angelika Böttger (Ludwig-Maximilians-University Munich) discussed the role of Notch signaling in *Hydra*, which functions in the re-establishment of head organizer activity and restricting tentacle patterning during head regeneration [26]. Böttger found that treating *Hydra* with the Notch inhibitor DAPT causes defects in head regeneration, budding, and nematocyte differentiation. An RNA-seq time course during DAPT recovery was used to identify putative direct targets of Notch. Bert Hobmayer (University of Innsbruck) discussed dissecting the function of secreted Frizzled-related proteins (sFRPs) during cnidarian development. To determine if sFRPs antagonize Wnt signaling in cnidarians, similar to their role in bilaterians, Hobmayer tested the expression pattern and function of *NvsFRP* in *Nematostella*. He found that *NvsFRP* is expressed in the aboral apical tuft region of the planula larva in a region that lacks Wnt expression. Furthermore, loss of aboral *NvsFRP* function disrupts apical tuft formation. Interestingly, *NvsFRP* can rescue axis duplication in *Xenopus* when Wnt is overactivated. However, the results of TOP-Flash assays, which are a read-out for β-catenin activity, do not indicate that *NvsFRP* acts as a Wnt inhibitor. Future work will aim to resolve this discrepancy and help clarify the role of secreted Frizzled in cnidarians. Hongyan Sun (graduate student, University of Miami) continued the discussion of Wnt signaling by sharing her comparative functional analysis of Axin between *Strongylocentrotus purpuratus* (purple sea urchin) and *Nematostella*. The canonical function of Axin is in the β-catenin destruction complex, where it directly binds to β-catenin and promotes its degradation. While the *S. purpuratus* Axin homolog has a β-catenin binding domain, the *Nematostella* Axin homolog does not. However, injection of *NvAxin* into both *S. purpuratus* and *Nematostella* embryos disrupts β-catenin nuclear localization. Thus, Sun suggests that additional scaffolding proteins must be involved.

Several talks highlighted gene function in relation to specific aspects of development, including genome activation, the formation of primordial germ cells (PGCs), metamorphosis, and regeneration. Febrimarsa (graduate student, National University of Ireland Galway) discussed DNA N^6^-Methyladenosine (6-mA) modification in *Hydractinia*. 6-mA DNA modification is a highly conserved feature from ctenophores to mammals, yet its biological function during animal development remains elusive [27–29]. Febrimarsa found a correlation between the loss of 6-mA and zygotic genome activation, similar to recent reports in zebrafish and mouse embryos. He found that 6-mA levels inversely correlate with transcriptional activity and proposed that 6-mA is a repressive mark that needs to be removed for genome activation. Cheng-Yi Chen (graduate student, Stowers Institute) investigated the specification and maturation of primordial germ cells (PGCs) in *Nematostella*. Vasa-positive PGCs form two clusters, one on each side of the pharynx of a primary polyp. These cells are localized right at the junction between *Hedgehog1* and *Patched* expression domains. Loss of function analysis for Hedgehog pathway members revealed a previously unappreciated role for this pathway in the specification and migration of *Nematostella* PGCs. Naga Nakanishi (University of Arkansas) performed CRISPR/Cas9-mediated mutagenesis of the GLW-amide peptide in *Nematostella*. Although these mutants did not display developmental defects, a significant delay in the timing of larval metamorphosis was observed, indicating that GLW-amide exerts control over life cycle transitions in *Nematostella* [30]. Jack Cazet (graduate student, University California, Davis) discussed his work investigating the transcriptional control of regeneration in *Hydra*. Cazet performed ATAC-seq experiments in regenerating head and foot tissue 3 h after injury. Cazet found that an identical generic wound response is activated in both tissues, which takes place largely in the absence of chromatin remodeling in regulatory regions. Thus, he proposed that in *Hydra* the injury response involves the activation of constitutively open regulatory regions. Finally, Ahmet Karabulut (graduate student, Stowers Institute) described his protocol for rapid delivery of shRNA into *Nematostella* eggs using electroporation [31, 32]. This approach is straightforward and easily scaled up for genetic screens, which could reveal novel genes required for cnidarian development.

## Technology: single-cell transcriptomics sheds light on developmental mechanisms

Rapid developments in genomic and transcriptomic technologies in recent years have enabled a deeper understanding of the genetic composition of cnidarians and of their gene-regulatory landscape. Single-cell RNA-seq (scRNA-seq), for instance, allows identification of rare cell types and transient cell states that occur during development or as stem cells differentiate. These approaches are widely applicable and provide an unparalleled opportunity for cnidarian researchers to uncover biological phenomena otherwise invisible using bulk tissue or mixed cell populations. Our technology speaker, Jeffrey Farrell (postdoctoral researcher, Harvard University), shared his expertise in single-cell transcriptomics by discussing his work examining zebrafish embryonic development. scRNA-seq allows for the measurement of single-cell transcriptional status while separating cells from their native spatial context. To address this issue, he introduced Seurat (v1.1), a computational method originally designed to retrieve spatial information of individual cells that he developed in collaboration with Rahul Satija (New York University) [33]. Farrell used Seurat to examine the expression levels of a set of landmark genes with known expression patterns to successfully infer the spatial location of individual cells from an scRNA-seq data set. Next, Farrell discussed his work reconstructing single-cell developmental trajectories of the first 12 h of zebrafish embryogenesis [34]. He sequenced thousands of embryonic cells over a developmental time course and established a simulated diffusion-based computational method called URD to reconstruct developmental trajectories. Finally, Farrell applied URD to build trajectories for 25 cell types and these data were illustrated in a stunning 3D branching tree.

Farrells’s talk demonstrated the power of scRNA-seq in a conventional model organism and it sparked spirited discussion. We then heard talks from two speakers who have successfully applied single-cell transcriptomics to cnidarian organisms. Stefan Siebert (University of California, Davis) discussed scRNA-seq data collected from *Hydra*. The adult polyp undergoes continuous cell turnover, thus providing a platform to study stem cell differentiation and tissue maintenance. By sequencing the transcriptomes of approximately 25,000 single cells, Siebert, in collaboration with Farrell, reconstructed the differentiation trajectories from the three lineages that make up the adult *Hydra*. The data suggest that neurons and gland cells share a common progenitor cell type in *Hydra*, a hypothesis that will be the subject of future studies [14]. Finally, the dataset allowed for the identification of molecular markers for many cell types, including previously elusive cell types such as neurons embedded in the endodermal layer. Flora Plessier, (graduate student, Pasteur Institute), presented progress applying scRNA-seq to *Nematostella*. The Marlow lab pioneered single-cell analysis in *Nematostella*, recently reporting the completion of a single-cell atlas covering both adult and larval stages [13]. Plessier will build upon this work in the Marlow lab using scRNA-seq to dissect neuronal cell differentiation trajectories during development and regeneration. To isolate subpopulations of neurons at different developmental stages, she first designed and generated a SoxC-kikGR transgenic reporter line. The sorted cells will then be prepared for scRNA-seq and subsequent data analysis. This targeted cell sorting approach, in conjunction with the cellular resolution obtained from scRNA-seq, will shed light on key neurogenic trajectories in *Nematostella*, and lay the groundwork for comparative studies with bilaterian animals.

## Neurobiology: bridging the gap between activity and function

Cnidarians are the closest group to the bilaterian clade that possesses a functional nervous system. Unlike bilaterian animals, cnidarians lack a highly centralized nervous system and instead exhibit complex behaviors using diffuse nerve nets [35, 36]. Understanding the full scope of the diversity and complexity of the cnidarian nervous system remains a formidable challenge. However, as highlighted in the two Cnidofest neurobiology sessions, cnidarian neurobiologists are tackling these questions with the help of cutting-edge technologies. To dissect the function of different neuronal subpopulations, Yukihiko Noro (King Abdullah University of Science and Technology) constructed several transgenic *Hydra* lines expressing GCaMP protein in different neuronal subtypes and performed calcium imaging. Hym-121 and Hym-355 positive neurons exhibited different excitation patterns. Noro hypothesized that Hym-121 positive neurons might function as mechanosensory neurons, as they responded strongly to mechanical stress applied using tweezers. Dylan Faltine-Gonzalez (graduate student, Lehigh University) investigated the mechanisms governing patterning of the *Nematostella* nervous system during embryogenesis. Previous studies have shown that the Notch and MEK signaling pathways play instrumental roles in patterning the *Nematostella* nervous system [37, 38]. To look for additional genetic cues, Faltine-Gonzalez performed shRNA-mediated gene knockdown of *NvAth*-*like*, a key regulator of neural differentiation. He then tested the expression levels of candidate downstream genes to identify potential targets that might play a role in specification of neuronal subpopulations. To test the function of these genes, Faltine-Gonzalez proposed to knock genes down in transgenic reporter animals to visualize the function of each gene in neuronal specification. Jamie Havrilak (postdoctoral researcher, Lehigh University) focused on the plasticity of *Nematostella* neurons during regeneration and starvation. Taking advantage of transgenic lines that express GFP in *NvLWamide*-positive and *SerumAmyloid*-*like*-positive neurons, Havrilak observed a positive correlation between the number of neurons along each mesentery and animal body size (Fig. 2b). She then applied a feeding–starvation regime, which either increased or decreased the body size, and demonstrated that the number of *NvLWamide*-*like* neurons scales according to the size of the animal [39]. Jonathan Lovas (graduate student, Columbia University) presented his work investigating neuronal circuit formation in *Hydra* from a unique perspective. Dissociated *Hydra* cells can reaggregate to ultimately form a normal animal. Lovas performed this procedure using a transgenic line expressing GCaMP in the neurons, which allows for the visualizations of nervous system activity by tracking calcium levels indicated by fluorescence [36]. Imaging of the aggregates at different stages demonstrated the phase transition from a ‘subcritical state’ in dissociated cells (in which activity is stochastic and rapidly reduces overtime) to a ‘supercritical state’ (in which the activity amplifies in circuits). Leslie Babonis (postdoctoral researcher, Whitney Laboratory for Marine Bioscience, University of Florida) interrogated the gene-regulatory network of cnidocytes, which have been suggested to share a common origin with neurons. Recent work in *Nematostella* demonstrated that *SoxB2*-positive progenitor cells give rise to both neurons and cnidocytes during embryogenesis [40], further supporting a common origin of cnidocytes and neurons. Babonis focused on transcription factors downstream of *SoxB2*. Using a double fluorescent in situ hybridization approach, she demonstrated that two of these genes, *PaxA* and *Mef2*, exhibit partially overlapping expression patterns, representing distinct cnidocyte lineages. She also tested the role of *Sox2* by generating knockout F0 animals using CRISPR/Cas9. She observed that these embryos exhibited reduced levels of the cnidocyte marker mcol-2, while undergoing normal neuronal development processes.

## Technology: using microfluidics to investigate nervous system function in small organisms

Microfluidic devices, which exploit microfabrication technologies to create integrated chips that can manipulate small volumes of fluid, have applications in cell sorting, next-generation sequencing, and imaging systems to facilitate scientific research. The small size of many cnidarian embryos, larvae, and even adults make them well-suited for use with this technology. Our technology speaker, Jacob Robinson (Rice University), has constructed customized microfluidic devices for small organisms to interrogate the connections between neural circuitry and behavior. According to Robinson, the idea of applying microfluidics first stemmed from the frustration over trying to perform electrophysiology in *C. elegans*. Due to the invasive nature of classic electrophysiology devices and the small size of the animal, previous experiments often utilized dissected worms, which limit the ability to relate neural recordings to animal behavior. To solve this problem, his lab designed microfluidic chambers, equipped with nanoscale silicon spikes for action potential detection. Using this device, he demonstrated long-term live imaging of *C. elegans* while simultaneously recording the behavior of the worm and the action potentials of a single muscle cell. Detailed behavioral and electrophysiological analysis revealed the presence of a sleep-like state, in which the worm remains inactive for prolonged periods. Customized microfluidics devices demonstrate a high level of flexibility to suit different purposes in different organisms. The overall scalability of these systems means parallel experiments and measurements are now possible.

Inspired by his success with worms, Robinson then applied a similar strategy to study *Hydra* nervous system activity and behavior. Adult *Hydra* polyps are comparable in size to adult *C. elegans*, while possessing a simpler tissue architecture, making them an attractive system for neurobiology research. Krishna Badhiwala (graduate student, Rice University) discussed how she and Robinson pioneered real-time behavior tracking in *Hydra* using customized microfluidics devices [41]. Badhiwala developed an automated image processing algorithm which captures body features such as the head and foot of a polyp across multiple frames of a time-lapse movie. This allowed her to retrieve positional information and subsequently dissect behavioral motifs, the combination of which results in complicated locomotion patterns. Interestingly, during long-term behavioral tracking, *Hydra* also exhibited periodicity in locomotion activity, reminiscent of the sleep-like state previously observed in *C. elegans*.

## Cell biology: examining the cellular dynamics underlying cnidarian morphogenesis

The relative simplicity of the cnidarian tissue architecture permits interrogation of cell biological processes that are fundamental to development and regeneration. In recent years, comparative analysis using cnidarian systems has provided new, and often surprising, insights into the diverse cellular programs employed during morphogenesis as well as tissue homeostasis [42–46]. Helen McNeill (Washington University in St. Louis) discussed the function of conserved cell–cell adhesion molecules Fat and Dachsous during *Hydra* development. These cadherins interact with each other and are crucial for the establishment of planar cell polarity (PCP) in bilaterian systems [47–49]. McNeill found that *Hydra* has one copy of a Fat-like gene (*HyFat*) and one Dachsous (*HyDs*), similar to *Drosophila*; both genes are ubiquitously expressed in epithelial cells. Using an antibody raised against HyFat, she then demonstrated that HyFat protein is distributed in a polarized fashion on the apical side of body column epithelium, suggesting a conserved role for *HyFat* in PCP establishment. In support of this, she demonstrated that knocking down *HyFat* resulted in severely deformed head structures. Tapan Goel (graduate student, University of California, San Diego) shared his fascinating story of *Hydra* mouth opening dynamics. *Hydra* tear their hypostome tissue every time they open their mouth. It is a process driven by cell shape changes with little or no cell rearrangement [50]. Goel noticed that mouth opening initiates with a fast opening phase, followed by a slower opening phase. Using a modified logistic equation, he was able to model the kinetics of the *Hydra* mouth opening process. He then discussed a series of perturbations he is performing, including using nerve-less *Hydra* and laser ablation of actin myonemes, with the goal of understanding the biology underlying his observations. In the final talk of the Cell Biology session, Taylor Skokan (graduate student, University of California, San Francisco) showed stunning live imaging of transgenic *Hydra* expressing Lifeact-GFP in ectodermal epithelial cells which revealed surprising actin dynamics. He observed large ring-shaped protrusions being formed on the apical surface of epithelial cells with extraordinary dynamics, reminiscent of macropinosomes found in mammalian macrophages. The formation of these structures can be robustly induced by the addition of Gd^3+^ ions and can be inhibited by applying pressure from the coverslip. The physiological purpose of these structures remains unknown, but this study nicely showcased the potential for application of advanced microscopy in cnidarian research.

## Cnidarian immunology: to kill, or not to kill, that is the question

Cnidarians have mechanisms to defend against microbial pathogens and distinguish between self and non-self, while often maintaining a beneficial symbiotic relationship with dinoflagellate symbionts [51, 52]. A deeper understanding of the cnidarian innate immune system will provide crucial insights into the early evolution of immunity. In this session, talks focused on using genetic and genomic approaches to address key questions of cnidarian immunology. Aidan Huene (graduate student, University of Pittsburgh) discussed her work investigating the genetic mechanisms underlying *Hydractinia* allorecognition, the process by which colonies decide whether to fuse or fight when they come into contact [22, 23, 53]. Two polymorphic transmembrane proteins, *Alr1* and *Alr2*, were previously identified as key players. Through careful observation, Huene realized that *Alr1* and *Alr2* alone are insufficient to explain all outcomes when two *Hydractinia* colonies interact. Colonies sharing identical *Alr1*/*Alr2* loci sometimes exhibit partial fusion, suggesting the presence of additional determinants. In collaboration with the Schnitzler and Baxevanis groups, Huene identified over 60 Alr-like genes within the same genetic complex near *Alr1* and *Alr2*. Through a series of genetic recombination experiments using outbred colonies, she mapped a third allodeterminant onto a 0.4-Mb genomic region. These results demonstrate that *Hydratinia* allorecognition relies on a more sophisticated molecular program than previously thought. Nikki Traylor-Knowles (University of Miami) presented progress in understanding the immune response of the coral *Pocillopora damicornis*. With collaborators, she sequenced and analyzed the genome and found that immune-related genes, such as components of the NF-κB pathway, were expanded in *Pocillopora*. She found that some immune-related genes are expressed in nematocytes, echoing recent findings in *Nematostella* [54]. Finally, to identify cell populations that are responsible for the immune response, Traylor-Knowles used a cell sorting assay, which revealed the presence of an ameboid cell type with phagocytotic activity. Leah Williams (graduate student, Boston University) presented her work on the function of Toll-like receptors (TLRs) in *Nematostella*. TLRs are a family of transmembrane receptor proteins that are capable of pathogen recognition in bilaterian animals. *Nematostella* possesses a single TLR gene that is structurally similar to the orthologous vertebrate gene. Using tissue culture systems, Williams first showed that NvTlr is able to activate the NF-κB pathway in human cell lines by recognizing heat-inactivated coral pathogen *Vibrio coralliilyticus* and bacterial flagellin protein. Next, using in situ hybridization and immunofluorescence, she found that nematosomes (motile bodies specific to *Nematostella*) are highly enriched for *NvTlr* and NF-κB components. Moreover, she demonstrated that nematosomes are capable of engulfing fluorescently labeled *V. coralliilyticus*. Genetic perturbations of *NvTlr* and *NvNF*-*κB* by morpholinos elicited developmental defects as well as failure of nematocyte specification. Taken together, these results suggest that innate immune signaling is functionally conserved in Cnidaria.

## Symbiosis: a closer look into cnidarian host–symbiont relationships

Many cnidarian species, especially corals and sea anemones, host intracellular symbionts: dinoflagellates of the family *Symbiodiniaceae*. Reef-building corals, for instance, cannot thrive in nutrient-poor tropical waters without nutritional assistance from their phototrophic symbionts. As emphasized by Weis in the keynote address, understanding the cnidarian–dinoflagellate symbiotic relationship is an urgent task, as global warming and seawater acidification are threatening the existence of coral species all over the world. The small sea anemone *Aiptasia* is widely used in laboratories as a model system to study cnidarian–symbiont interactions because unlike corals, *Aiptasia* can be easily cultured and manipulated in a laboratory setting. Lorraine Ling (postdoctoral researcher, Stanford University) investigated the role of C-type lectins in host–symbiont recognition using *Aiptasia*. C-type lectins are a group of carbohydrate-binding proteins that facilitate cell–cell adhesion, immune response, and pathogen detection. Ling found that two C-type lectins, *Ctl1* and *Ctl6*, are downregulated during symbiont establishment. Next, using in vitro binding assays, she found that Ctl1 recombinant protein binds strongly to incompatible strains, indicating that C-type lectins might contribute to the specificity of host–symbiont recognition. Thomas Gilmore (Boston University) focused on the dynamics of the NF-κB pathway during symbiont establishment in *Aiptasia*. Gilmore found that NF-κB signaling activity increases upon depletion of symbionts and decreases upon introduction of compatible symbionts to naïve *Aiptasia*. Symbiotic *Aiptasia* were more susceptible to pathogenic bacterium, which may explain why lower levels of bacterial infection were found in recently bleached corals. Taken together, these data support the hypothesis that symbionts partially suppress the host immune system and may compromise the ability of the host to respond to foreign pathogens or environmental stress. Casandra Newkirk (graduate student, Whitney Laboratory, University of Florida) investigated the potential connection between symbiont establishment and life stage transition in the upside-down jellyfish, *Cassiopea xamachana*. The acquisition of algal symbionts at the polyp stage is a prerequisite for the metamorphosis and subsequent strobilation. Newkirk generated asymbiotic polyps and tested the ability of different strains of symbionts to grow in the polyp and trigger strobilation. Among the three strains tested, strain A194 exhibited the slowest growth and slowest trigger for strobilation. However, the timing of strobilation did not correspond to the absolute number of symbionts within a host polyp, indicating the presence of additional signals. The final speaker of the session, Juris Grasis (University of California, Merced), argued that viruses, just like bacteria and eukaryotic symbionts, are an inseparable component of the animal holobiont. Using *Hydra* as a model system, Grasis surveyed the *Hydra* virome with sequencing methods [55]. Taking advantage of this dataset, the Grasis lab is further exploring the diversity of *Hydra*-associated viruses and their function during host immune response. Interestingly, Grasis observed a strong immune response when introducing foreign viruses into *Hydra*, even under germ-free conditions, suggesting that *Hydra* possess an underappreciated viral discrimination mechanism.

## Ecology: how cnidarians face environmental challenges

Cnidarians, such as corals and sea anemones, form the foundation for some of the most diverse and complex ecosystems in the world. Exploring the interactions between cnidarians and their living habitat provides crucial information to help ensure their survival in the everchanging environment. Whitney Leach (graduate student, University of North Carolina at Charlotte) explored the molecular nature of the circadian clock in *Nematostella*. Previous studies have demonstrated the existence of behavioral, transcriptional, and metabolic rhythmicity in *Nematostella* polyps subjected to diel light cycle [25, 56–58]. To explore the molecular response in detail, Leach compared transcriptomic profiles in light cycle entrained animals both before and after light removal. She observed that some previously known oscillating genes, such as *NvPar*-*bZIPC*, stopped cycling in response to light removal while others, such as *NvCry1a*, remain oscillating. Interestingly, even at 48 h post-light removal, oscillation patterns were observed in approximately 180 different genes, suggesting the presence of a persistent circadian rhythm. Hanny Rivera (postdoctoral researcher, Woods Hole Oceanographic Institution) used *Nematostella* to better understand the genetic inheritance of thermal tolerance. She found that *Nematostella* populations in the wild are subjected to a large range of daily temperature fluctuations. She therefore tested in the laboratory the thermal tolerance of *Nematostella* planula larvae produced from parents reared in two different temperature conditions: (1) constant temperature and (2) intermittent heat shock. She observed a possible inheritance of thermal tolerance that may be transmitted maternally. Finally, Rivera proposed to test *Nematostella* strains collected from different geological locations to better understand the inheritance and adaptation of thermal tolerance in wild populations. Sérgio Stampar (São Paulo State University) discussed the advantages of using the traditionally understudied tube anemone, *Ceriantharia*, as a model system to study the influence of the environment on animal life stages. By refining the life stage descriptions of *Ceriantharia*, Stampar and his collaborators revealed remarkable diversity and plasticity in the development of these animals, such as the presence or absence of a planula larvae stage. Depending on nutritional condition and additional environmental cues, a single *Ceriantharia* species can remain as a planktonic larva for a variable amount of time, ranging from a week to 3 months. This extreme flexibility during development, in addition to other unique biological traits, such as a linearized mitochondrial genome and abnormal toxin assemblage, makes *Ceriantharia* an attractive model system for future cnidarian research [59]. Finally, Grace Snyder (graduate student, University of Miami) discussed developing new biomarkers to understand the diversity of cell types in *Pocillopora* corals. She performed a screen to identify biomarkers that could be used to isolate coral cells by FACS. She found that coral cell types had differential binding affinities to certain dyes and that autofluorescence from dinoflagellate symbionts could be used to isolate gastrodermal cells.

## Evolution: phylogeny of the past, perspectives of the future

As a sister group to bilaterians, cnidarians are keys to understanding evolutionary transitions, such as segregation of the mesoderm, bilateral symmetry, and nervous system centralization. Cnidarians share many conserved genes and signaling pathways with bilaterians, so these transitions must largely result from differential use of the same genetic toolkit. Natasha Picciani (graduate student, University of California, Santa Barbara) presented her recent findings regarding the evolution of eyes in cnidarians [60]. Many cnidarian species possess photo-sensing organs with a range of tissue complexity and image-forming abilities. Picciani surveyed diverse cnidarian lineages, constructed a phylogenetic tree, and then mapped the presence of eyes (defined by photoreceptor cells adjacent to pigment cells) onto this tree. From these data, she hypothesized that eyes independently evolved in Medusozoa multiple times. To test this, she built a large opsin phylogeny, which also supports multiple origins of cnidarian eyes. E. Sally Chang (graduate student, University of Kansas) presented her work investigating how the invasive hydrozoan *Cordylophora caspia* moved from a strictly estuarine environment to freshwater. Multiple subspecies tolerate different salinity ranges, presenting a perfect opportunity to investigate the marine to freshwater transition. Using RAD-seq methods, Chang built a phylogeny of *Cordylophora* collected from different geological locations with different salinities. By mapping the native salinity on to the phylogenetic tree, she demonstrated that lineages are separated by salinity. Ancestral character state reconstruction revealed that the *Cordylophora* ancestor may already have possessed the ability to tolerate freshwater. Adolfo Lara (graduate student, American Museum of Natural History) presented his work on the phylogenetic distribution of gap junction proteins in Cnidaria. By surveying multiple cnidarian species, including understudied branches such as Staurozoa, Myxozoa, and Cubozoa, Lara found that pannexins are enriched in anthozoan species such as *Nematostella*, while innexins are exclusively found in medusozoans. How this impacts the function of gap junctions in these clades requires further investigation. Meg Daly (Ohio State University) presented her work exploring the evolutionary relationships within the anthozoan lineage. She first determined the relationships between major anthozoan lineages using highly conserved DNA sequences, and subsequently relied on variable genes to determine relationships within each lineage. To add additional species in the absence of genomic data, Daly used morphological analysis to manually determine position on the tree. Her phylogeny suggests that the presence of symbionts is a rare trait in anthozoans that independently evolved multiple times. Last, Lauren Vandepas (graduate student, University of Washington) analyzed the evolution and function of chitin synthase across Cnidaria. Taking a genomics approach, Vandepas identified chitin synthase genes in several cnidarian genomes and using an affinity tag for chitin she found that chitin is indeed present in the tissues of scyphozoan, hydrozoan, and anthozoan animals. In *Nematostella*, for instance, chitin is found in spirocytes (a type of stinging cell) and in the mesoglea. Chitin is a common biomolecule that provides structural support for animals such as arthropods and crustaceans, but a function in cnidarians is unexplored. Vandepas proposed that cnidarians might use chitin to provide additional structural support.

## Poster sessions and awards

A total of 43 posters were displayed in two poster sessions during the meeting. Before each poster session, all poster presenters gave a 2-min lightning talk to advertise their research to all meeting attendees. According to post-meeting survey results, the lightning talks were effective and well received. The posters covered diverse topics of cnidarian biology, including genomics and transcriptomics, regeneration and stem cell biology, behavioral biology, neuroscience, ecology, and evolution. Due to limitations of space, we will not be able to discuss all of the exciting work presented during the poster sessions. A few highlights include: Bob Zimmermann (postdoctoral researcher, University of Vienna) presented the community effort to generate a chromosome-level genome assembly of the starlet sea anemone *Nematostella vectensis*; Paige Zhang (graduate student, University of California, Los Angeles) probed the DNA methylation pattern of the moon jelly, *Aurelia* sp.; Hiroshi Shimizu (King Abdullah University of Science and Technology) presented a potential gravity sensing mechanism in *Hydra*; Bryan Teefy (graduate student, University of California, Davis) discussed the function of the PIWI-piRNA pathway in *Hydra* to repress transposons in somatic stem cells.

Among the many outstanding presentations and posters, Ahmet Karabulut (graduate student, Stowers) and Taylor Skoken (graduate student, UCSF) won the best oral presentation awards; Jennifer Spillane (graduate student, University of New Hampshire) and Shelcie Menard (postdoctoral researcher, University of North Carolina Charlotte) won the best lighting talk awards; and Abby Primack (graduate student, UC Davis) and Rui Wang (graduate student, Swarthmore College) won the best poster awards.

## Summary

As emphasized by Virginia Weis in her keynote address, it takes a village. It takes community effort, not only to advance coral symbiosis studies, but also to push forward the entire field of cnidarian research. At this meeting, the emergence of a bright new generation of cnidarian biologists was on display and is evidence that our community is growing. The development of new technologies that can be readily applied to cnidarians is flooding our field with new and exciting ideas. This US-based meeting will be held on a biennial basis, alternating between east and west coasts to balance travel burdens. The next meeting will be held at the University of California, Davis on September 9–13th, 2020.
